# Attitudes towards and impact of letters of recommendation for anesthesiology residency applicants

**DOI:** 10.1080/10872981.2021.1924599

**Published:** 2021-05-07

**Authors:** Carl E. Jn Pierre, Garret M. Weber, Apolonia E. Abramowicz

**Affiliations:** a New York Medical College, School of Medicine, Valhalla, New York; b Department of Anesthesiology, New York Medical College, Westchester Medical Center, NY, New York

**Keywords:** Letters of recommendation, program directors, associate/assistant program directors, anesthesiology residency program application

## Abstract

**Background:** This survey aims to identify the relative value and the critical components of anesthesiology letters of recommendation(LORs) from the perspective of Program Directors (PDs) and Associate/Assistant Program Directors (APDs). Knowledge and insights originating from this survey might add to the understanding of the anesthesiology residency selection process and mitigate unintended linguistic biases.

**Methodology**: Anonymous online surveys were sent to anesthesiology PDs/APDs from the Accreditation Council for Graduate Medical Education (ACGME) accredited anesthesiology residency Programs in the USA (US), as listed on the ACGME website and the American Medical Association Fellowship and Residency Electronic Interactive Database (AMA FREIDA) Residency Program Database. The survey authors were blinded to the identity of the respondents.

**Results**: 62 out of 183 (33.8%) invited anesthesiology PDs/APDs completed the survey anonymously. In our survey, LORs are reported as more important in granting an interview than in making the rank list. 64% of respondents prefer narrative LORs. 77.4% of respondents look for specific keywords in LORs. Keywords such as ‘top % of students’ and ‘we are recruiting this candidate’ indicate a strong letter of recommendation while keywords such as ‘I recommend to your program’ or non-superlative descriptions indicate a weak letter of recommendation. Other key components of LORs include the specialty of the letter-writer, according to 84% of respondents, with anesthesiology as the most valuable specialty. Although narrative LORs are preferred, 55.1% of respondents are not satisfied with the content of narrative LORs.

**Conclusion**: LORs containing specific keywords play an important role in the application to anesthesiology residency, particularly when submitted by an anesthesiologist. While narrative LORs are still the preferred format, most of our respondents feel they need improvements. The authors suggest specific LOR improvements including creating formalized LOR training, adding a style guide, and applying comparative scales, with standardized vocabulary in the narrative LOR.

## Introduction

Letters of recommendation (LOR) have long been a part of the application process to residency programs[1]. Although LORs are an imprecise predictor of students’ future performance during residency[2], they are a major part of an application. In Anesthesiology, graduation from a USA (US) medical school, higher USA Medical Licensing Examination (USMLE) step 1 and 2 scores, younger age and female gender have previously been identified as characteristics associated with successful admission to a residency[3]. However, no prior study has attempted to identify the relative value of LORs in the application to an anesthesiology residency program from the perspective of the Program Directors (PDs). In other specialties, specific components such as personalization of letters and academic rank of the writer have been shown to impart value to LORs[4]. Conversely, other studies have identified characteristics as well as cliché phrases such as ‘If I can provide any additional information please call … ’ that can negatively impact the value of LORs[5].

Currently, many medical schools have adopted a pass/fail system for pre-clinical years, which has been shown to improve the psychological well-being of medical students[6]. With the announcement that USMLE program will change the Step 1 score reporting from a three-digit numeric score to Pass/Fail only [7], LORs may become more important to identify ‘good fit’ anesthesiology residency candidates.

This survey aims to identify the components of LORs that PDs/APDs, i.e., those primarily responsible for resident selection, consider valuable in an anesthesiology residency application. Knowledge and insights into LOR originating from this study could possibly improve the selection process and mitigate unintended linguistic biases [8] triggered by LOR content in anesthesiology residency Program application review and categorization.

## Methodology

This study was deemed minimal risk and exempt from consent after New York Medical College/Westchester Medical Center (NYMC/WMC) Institutional Review Board (IRB) review # 12,995.

We designed a survey to explore four main areas: 1) Relative weight of LORs, 2) Key elements in LORs, 3) Importance of the writer’s rank and specialty, 4) Format of LORs. The survey utilized Likert scale and free text qualitative responses, which were recorded through Qualtrics software; the survey was administered over a period of 3 months. Our survey instrument is available for review in entirety through Supplemental Appendix. Anesthesiology PDs and APDs were identified in July 2019 by review of the current Accreditation Council for Graduate Medical Education (ACGME) accredited anesthesiology residency programs in the US on the public website of the ACGME, and the American Medical Association Fellowship and Residency Electronic Interactive Database (AMA FREIDA) residency program Database, and by emailing individual Program coordinators to obtain contact information of PDs and APDs. The survey authors were blinded to the identity of the respondents. A restriction was placed on Qualtrics prior to sending the surveys, limiting the survey submission to one per person. Therefore, respondents who had previously completed the survey were unable to access it a second time.

Only current anesthesiology PDs and APDs of ACGME accredited anesthesiology residency programs were included in this study. Both FREIDA [9] and ACGME websites [10] identified 151 accredited programs as of July 2019. PDs and APDs from international-ACGME (I-ACGME) accredited programs were not included.

A thematic analysis, using constant comparative method, was used to analyze the qualitative data. All answers, including specific keywords were recorded. Common qualitative responses were grouped prospectively and reported in this study.

## Results

### Overall response rate

**Figure 1.**
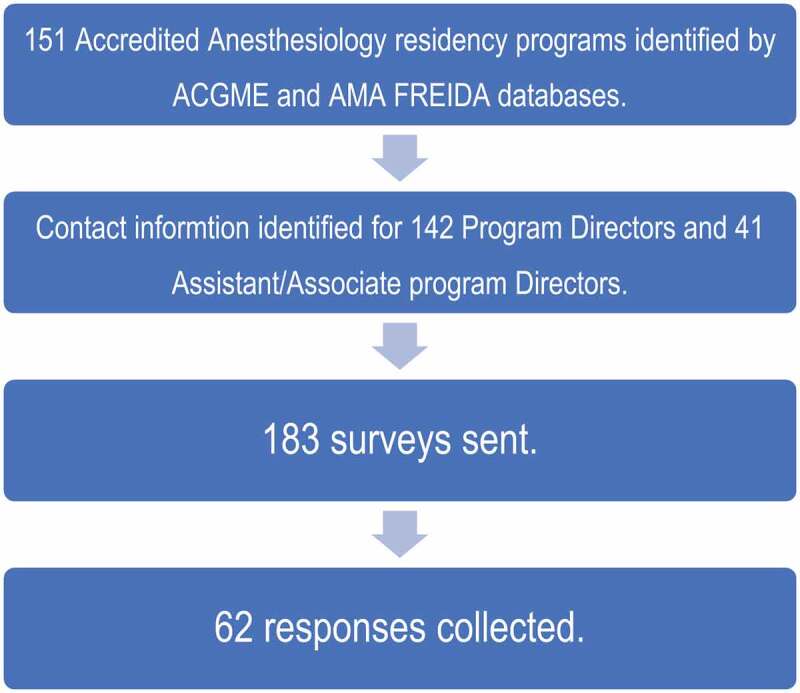
Total number of responses identified by ACGME and AMA FREIDA databases

**Figure 2.**
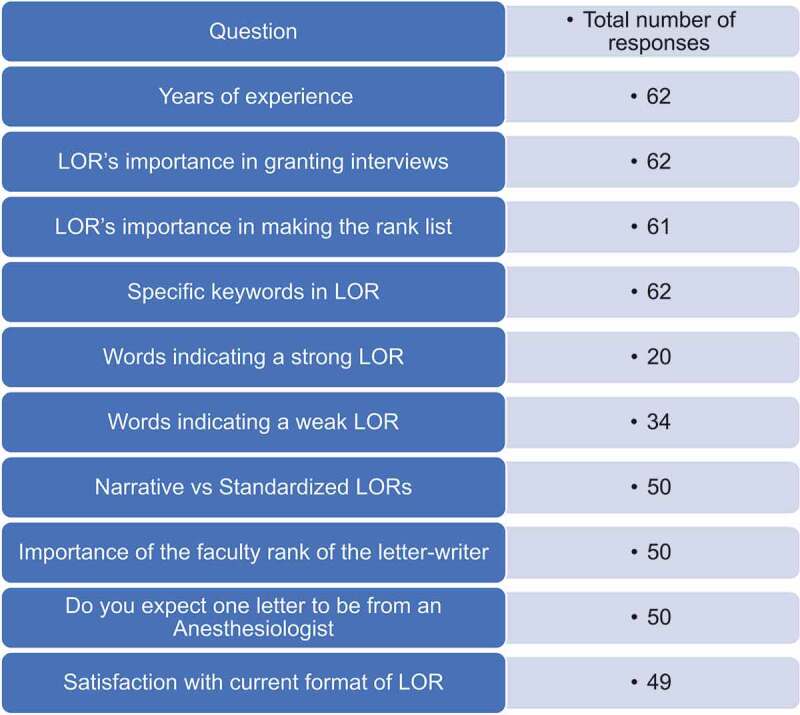
Number of respondents that answered individual questions

Out of 151 anesthesiology residency programs identified in July 2019, we obtained/confirmed 142 PDs’ and 41 APDs’ contact information via AMA FREIDA and by contacting anesthesiology residency Program Coordinators. The survey was thus emailed to 142 anesthesiology PDs and 41 APDs (N = 183). A total of 62 out of 183 anesthesia PDs and APDs surveyed completed the survey anonymously (Figure 1). It should be noted that not all respondents answered the survey in its entirety, and therefore the number of respondents may be different from one question to the next; the total responses appear in each table. Our overall response rate was 33.8%. The individual survey question response numbers appear in Figure 2. Years of experience in the role of PD/APD were recorded in Table 1.

**Table 1.** Years of experience of respondents (PDs and APDs)

Years of Experience	%	N
0–5 years	43.55%	27
5–10 years	37.10%	23
10–20 years	14.52%	9
>20 years	4.84%	3
Total	100%	62

### Relative importance of LORs in application status

We first attempted to determine the relative weight of the LORs, in both granting an interview and generating the rank order list.

53.23% (n = 33) of respondents reported that the LOR is moderately to very important in granting an interview, while 46.78% (n = 29) reported that it is only slightly or not important at all (Table 2).

**Table 2.** Importance of LOR in granting interview and in making rank list

	Very important	Moderately important	Slightly important	Not at all important	Total
Influence of LOR on interviews	11.29% (n = 7)	41.94% (n = 26)	43.55% (n = 27)	3.23% (n = 2)	100% (n = 62)
Influence of LOR on rank list	9.84% (n = 6)	31.15% (n = 19)	44.26% (n = 27)	14.75% (n = 9)	100% (n = 61)

When creating the rank order list however, only 40.99% (n = 25) considered it moderately to very important, while 59.01% (n = 36) of respondents indicated that LORs are either slightly or not important at all (Table 2).

77.4% (n = 48) of survey respondents affirm they look for specific keywords in an LOR (Table 3).

**Table 3.** Are there keywords you look for when reviewing LORs?

Answer	%	n
Yes	77.42%	48
No	22.58%	14
Total	100%	62

### Key words in a LOR

Which words indicate a strong LOR?

To 41.67% (n = 20) of respondents, ‘top % of students’ indicates a strong LOR. 39.58% (n = 19) listed ‘we are recruiting this candidate’, 16.67% (n = 8) listed ‘outstanding’, 14.58% (n = 7) cited ‘best’, 10.41% (n = 5) listed ‘highest recommendation’, 6.25% (n = 3) listed ‘hardworking’, ‘technical skill progression’, or ‘would allow (candidate) to take care of own family’, ‘receptive to feedback’, ‘uplifting’, ‘superb’, ‘team player’, ‘without reservation’, ‘strong’ or ‘excellent fund of knowledge’ were each listed by 4.16% (n = 2) respondents. Other favorable keywords include ‘compassionate, adaptable, accommodating, excellent, situational awareness’.

54.83% (n = 34) of respondents also reported words they believe indicate a weak LOR.

41.17% (n = 14) of these respondents mentioned ‘good’ as indicative of weakness. 20.59% (n = 7) reported the word ‘strong’ as a potential red flag, and 14.7% (n = 5) reported the word ‘solid’ as having a negative connotation. ‘I recommend to your program’, non-superlative descriptions and excerpts from Curriculum Vitae (CVs) were also noted to reduce the strength of LOR by 8.82% (n = 3) of respondents. Other words that were listed as weak descriptors include ‘adequate’, ‘poor’, ‘bottom x %’, ‘on par’, ‘performed satisfactorily’, ‘tried hard’, ‘average’, and ‘I recommend (without an adverb).’ A complete list of words is listed in the Supplementary Appendix.

We then asked the remaining 22.58% (n = 14) respondents who indicated that there were no specific keywords they consider as adding value to a LOR (Table 3), what highlights a LOR?

28.57% (n = 4) of these respondents reported that a personal LOR makes more of an impression than a generic/impersonal letter. Meanwhile, 2 other respondents (14.28%) mentioned an interest in the academic title of the letter-writer, as well as specific anecdotes about the residency candidate, as reflecting a positive LOR. Specific anecdotes cited by some of our respondents include ‘someone who came in early to place all the IVs’, or ‘someone who looked up every drug we used and was able to discuss the pharmacology, side effects and appropriate applications intelligently’. The other responses included specific examples of interactions highlighting the residency candidate’s qualities, description of the nature of the relationship between the writer and the residency candidate, and academic rank of the letter-writer.

### Preference of narrative versus standardized letter format

We asked survey respondents if they have a preference between narrative and standardized LORs (SLORs), and why.

50 respondents responded to this question, with 36% (n = 18) expressing a preference for SLORs.

Out of these 18 respondents, 38.89% (n = 7) reported that the standardized format, as opposed to a narrative LOR, makes it easier to compare candidates. 27.78% (n = 5) cited efficiency as the reason for their preference, stating that it is easier to read multiple LORs when they are in the standardized format. 16.67% (n = 3) reported that SLORs are more objective, and 16.67% (n = 3) favored the consistency of SLORs. Other reasons why respondents preferred SLORs included the perception that they are easier to interpret and that ‘narrative LORs are more open to bias’. SLOR also ‘forces letter-writers to rank the applicants in tiers’.

64% (n = 32) of respondents, however, expressed a preference for narrative LORs. According to 31.25% (n = 10) of them, narrative LORs portray a more accurate profile of the residency candidates, allowing letter-writers to express their opinion and add specific information or personal anecdotes. Narrative LORs also demonstrate the nature of the relationship between the writer and the residency candidate (according to 18.75% [n = 6]) and are more personalized (15.625% [n = 5]).

### Importance of the author letter-writer’s profile

Next, our respondents were asked, 1) How significant is the faculty rank of the letter-writer? and 2) How important is the specialty of the letter-writer?

To each of these two questions we received 50 responses, which were ranked using a Likert scale from very important to not important at all. Table 4 list the answers to each of these questions separately.

**Table 4.** Importance of the faculty rank and the specialty of the letter-writer

	Very important	Moderately important	Slightly important	Not at all important	Total
Influence of faculty rank of the letter-writer	6.00% (n = 3)	40.00% (n = 20)	36.00% (n = 18)	18.00% (n = 9)	100% (n = 50)
Influence of the specialty of the letter-writer	40.00% (n = 20)	44.00% (n = 22)	12.00% (n = 6)	4.00% (n = 2)	100% (n = 50)

46% (n = 23) of respondents believe that faculty rank of the letter-writer is moderately to very important, while 54% (n = 27) believe that it is slightly important to not important at all.

Table 4 shows that 84% (n = 42) believe that the specialty of the letter-writer is moderately to very important. We asked these respondents to list the top 5 specialties that they consider most valuable, and to rank these specialties from 1 to 5, with 1 being the most valuable, and 5 being the least (5^th^ most) valuable on a Likert scale.

Most valuable (1 on Likert scale):

92.85% (n = 39) of respondents listed anesthesiology as the most valuable specialty, while 4.76% (n = 2) of respondents listed Internal Medicine as the most valuable.

second most valuable (2 on the Likert scale):

Surgery was listed as the second most valuable specialty by 33.33% (n = 14) of the respondents while 26.19% (n = 11) chose Internal Medicine, and 16.67% (n = 7) chose Critical Care.

Other specialties that were listed less frequently and were ranked lower than the top 2 on the Likert scale, for example, 3^rd^, 4^th^, or 5^th^, include Family Medicine, Psychiatry, Pulmonology, Cardiology, OB/GYN and Pediatrics.

98% of respondents expect at least one letter to come from an anesthesiologist.

We then proceeded to ask the respondents why they expected at least 1 letter from an anesthesiologist:

32% (n = 15) of our respondents reported that providing a LOR from an anesthesiologist shows that the residency candidate is interested in and committed to the field of anesthesiology, rather than treating anesthesiology as a ‘back-up plan in the Match’, as stated by one of the respondents. Another benefit is that it shows that someone from the specialty has ‘laid eyes on the residency candidate’, as stated by 23.4% (n = 11) of the respondents. LORs from anesthesiologists also demonstrate that the candidate has had ‘some exposure to the field’, according to 23.53% (n = 12) of respondents. Furthermore, applying to an anesthesiology residency without a single letter from an anesthesiologist is a ‘liability’, according to 10.64% (n = 5) of respondents.

### Recommended changes to format of LOR

Table 5 depicts how satisfied respondents are with the quality, format and information content (‘merit’) of LORs. 55.1% (n = 27) expressed dissatisfaction with the ‘merit’ of LORs. Subsequently, we asked them what specific changes to LORs they recommend?

**Table 5.** Are you generally satisfied with the caliber, format and content of current LORs?

Answer	%	
Yes	44.90%	22
No	55.10%	27
Total	100%	49

Responses varied highly. 14% (n = 7) of respondents would prefer SLORs. Other suggested changes include shorter, more focused letters, inclusion of objective data such as a ranking system and specific descriptions of the letter-writer’s interaction with the residency candidate.

## Discussion

LORs are an integral part of the residency and fellowship application process[11]. They serve as a highly coded form of interprofessional communication. However, the impact of LOR on PDs’ and APDs’ decisions on the anesthesiology candidates’ application ranking is unclear. This study aims to assess the value that LORs bring to the anesthesiology residency application process, as well as to analyze the desirable components and structure of LORs from the perspective of anesthesiology PDs.

### Overall importance of LORs

Our survey indicates that LORs are more important in granting an interview than in generating the rank order list; a finding supported by the 2018 NRMP Program Director survey, which listed LORs as the second most important factor in granting an interview, and as the 6^th^ most important factor in making the rank list in any specialty[12].

### Role of keywords in LORs

Our results also indicate that particular keywords and phrases are highly valued. According to our respondents, the most preferred phrase is ‘top x % of students’. Conversely, most respondents dislike SLORs even though they include tiering. Additionally, descriptors with superlatives such as ‘I recommend without reservation’ and comments such as ‘we are recruiting this candidate’, are preferred. Meanwhile, the absence of superlative descriptors such as no adverb to follow ‘I recommend’, is viewed as a red flag. The importance of keywords is further highlighted in surgery LORs where similar key phrases such as ‘we will plan to recruit this candidate’, and ‘I give my highest recommendation’ were highly valued [13].

The use and interpretation of particular keywords and phrases may represent a coded language between anesthesiology letter-writers and PDs/APDs. The implications of this are concerning. Our results indicate that the coded language does matter, and we speculate that misinterpretation of the message may have unintended consequences for the residency candidate. Prior studies in general surgery residency LORs have described a hidden or indirect LOR code as means of describing and stratifying residency candidates. The lack of use of particular phrases was detrimental to the surgical residency candidate [14]. While the direct consequence of coded language to the anesthesiology residency candidate has not been specifically studied, one can infer that it may have similar consequences. Coded LOR language that is rarely defined becomes difficult to decipher and prone to misinterpretation[14]. Wright et al. has suggested that inclusion of clear, ‘uncoded’ comparative language which allows for ranking of residency candidates may improve the quality of narrative LORs and allow for transparent and meaningful communication from letter-writer to evaluator [15].

Furthermore, our results indicate that there may be differences in the interpretation of specific keywords. For instance, ‘strong’ has been noted as both a red flag and praise by our respondents. In this respect, the intended message from the letter-writer may differ from what is received by the evaluators. These keywords and phrases may also be categorized based on an unofficial hierarchy. For instance, in surgery LORs [13], the phrase ‘One of the best’ is considered superlative to ‘Outstanding’, which in turn trumped the word ‘excellent’. As such, unfamiliarity with this classification system may cause inexperienced letter-writers to convey a message different than intended. This highlights the lack of standardization in the inclusion of various keywords and phrases as a potential limitation to the current free-form narrative LOR format[16].

### Ideal format of LORs

Between 1997, and 2017, several specialties including Emergency Medicine, Otolaryngology, Dermatology and Plastic Surgery have transitioned to a standardized LOR format. Standardized LORs were introduced to simplify LORs, minimize of the ‘variance between applicants’ attributes’ and reduce superlatives bias often seen in narrative LORs [17]. This switch has brought on mixed results, with reports of improved inter-rater reliability, more efficient interpretation of LORs time, and reduced gender bias [17], but also reports of grade inflation, which fails to mitigate the superlative inflation seen in narrative LORs [13,17]

Despite the fact that other specialties have moved toward standardized LORs [2,15,18–21], most of our respondents (64% [n = 32]) showed a preference for narrative LORs. A similar finding has been observed among surgery PDS and APDs [14]. LORs provide a unique platform for the assessment of otherwise difficult-to-measure characteristics that are important in selecting a candidate. Additionally, narrative LORs have the benefit of describing the relationship between letter-writer and residency candidate, as stated by 18.75% (n = 6) of respondents. Narrative LORs generally include a brief introduction which describes the aforementioned relationship[22]. However, the importance of the description of the nature of this relationship is conflicting in prior studies. For instance, a narrative description of the letter writer and applicant relationship was found to be extremely valuable in Dermatology residency candidates[4], but was found to be less important in general surgery residency candidates[13].

Although narrative LORs are preferred by the majority of our respondents, 36% (n = 18) of them expressed a preference for SLOR, and 55.1% (n = 27) of expressed dissatisfaction with the current format of the narrative LOR. One possible explanation of the dissatisfaction with the current format may be its potential for creating misinterpretation of the LOR. The subjective nature of the narrative format allows for different interpretations by the readers. For instance, the presence and choice of superlative adjectives in the description of the residency candidate may falsely influence the reader’s interpretation, as observed by Morgenstern et al [23]. This supports our findings that the presence of non-superlative descriptors such as ‘I recommend (without an adverb)’ may have a negative impact on the candidates’ application to residency, thus demonstrating that the message received by PDs may be vastly different from one intended by the letter-writer.

### Letter-writer’s profile

The content of LORs varies among letter-writers. However, common factors valued by our respondents include the specialty of the letter-writer. Virtually all (98% [n = 49]) the respondents believe that at least one letter should come from an anesthesiologist. Additionally, 92.85% (n = 39) of respondents consider LORs written by anesthesiologists as the most highly valued. Respondents believe that they affirm the residency candidate’s commitment to the field of anesthesiology. They are also used as a source of information about the residency candidate’s aptitude for the field. Among other specialties of the letter-writers’, the second most highly ranked and valuable ones, according to our results, include Surgery, Internal Medicine, and Critical Care. This may be a reflection of Anesthesiology’s wide scope of practice, which includes not only perioperative care but also trauma, critical care, and medical and surgical acute care emergencies[24].

Lastly, the faculty rank of the letter-writer was rated slightly to not important at all by the majority of respondents. We conclude that the content of LORs may be more important than the academic rank of the letter-writer. This finding is supported by Naples et al. who observed that surgical PDs, and Clerkship Directors did not find much value in a surgical Chair’s letter [14]. Surgical PDs and Clerkship Directors stated that the Chairs generally do not write the letter themselves, and that it is often generic and does not provide meaningful information. According to their data, the generic content of LORs from Chairs or ‘big-name person’ is much less valuable than a LOR from a letter-writer who ‘knows the candidate the best’[14]

We may also conclude that the academic profile and name recognition of the letter-writer may be more important than their academic rank. This finding is observed in Plastic Surgery, as PDs/APDs greatly value LORs from ‘well-known, highly published faculty’[17].

### Proposed improvements to the LOR

Based on the survey results, we suggest several areas of improvements to the current free-form, narrative LORs as described below. However, further research is needed on to how to incorporate our results into practice.

55.1% (n = 27) of our respondents state that they are not satisfied with the current format of the narrative LORs. Though other specialties have moved towards SLORs, only 36% (n = 18) of our respondents expressed a desire to switch from narrative LOR to SLOR.

Comparative Ranking Classification:

Current SLORs have a limited narrative component with word count restrictions [25,26]. We propose to continue the traditional narrative LOR with the addition of some elements of SLORs such as a Likert scale rating and identifying core-valued traits in a residency candidate. This Likert scale would allow for direct comparison of residency candidates, resulting in more objective data.

Addition of a Style Guide

Although guides to LOR writing already exist, they are generic and not tailored to fit the needs of specific specialties[27]. These style guides include recommendations on the organization of the LOR, and information from the residency candidates helpful in crafting the LOR; however, they fail to indicate what makes a strong or a weak LOR in the domains of choice of specific keywords or phrases [28]. We therefore recommend the creation of a style guide tailored for anesthesiology and its promulgation among the stakeholders.

An anesthesiology-specific style guide for letter-writers may help reduce ambiguity in the writing and interpretation of narrative LOR. This style guide could include a list of recommended descriptors, keywords and phrases indicating gradation of the strength of the LOR. The addition of such a list could be highly valuable, as highlighted by Kong J. et al. who stated that the use of strong descriptors associated with traits of the residency candidate greatly enriches a LOR[29].

It may also provide clarity as to the intended meaning of letters for the evaluators and readers of LORs. Further research is needed to determine which specific phrases and keywords would be important in an anesthesiology LOR style guide.

Training of letter-writers

Formalized training for the writing and interpretation of the LOR may also be beneficial. Further research is needed to determine the type and method of training of letter writers, which may improve communication between letter-writers and readers, while mitigating unconscious biases based on grammar, word choice, syntax, or other non-content factors that may influence the perception of the letter. According to Chopra et al., subspecialty medicine PDs/APDs understand the value of specific components of LORs, and therefore include such components much more consistently when writing LORs. In contrast, LORs from elective rotation supervisors are of the lowest quality. This finding was illustrated in Chopra’s 5-point composite endpoint, which compared LORs based on the inclusion of 5 components deemed necessary for a LOR to be considered high-quality. They have found that 62.1% of subspecialty medicine PDs/APDs’ LORs included at least 4 out of 5 components, while none of the LORs from elective rotation letter-writers included more than 3 components [30]. It is possible that PDs and APDs incorporate into LORs information that they themselves most value. LORs from PDs and APDs are of much higher quality compared to LORs from non-PDs/APDs and letter-writers from elective rotations. PDs/APDs may serve as the most ideal trainers for letter-writers and future letter evaluators[30].

## Study limitations

Limitations to this survey include a relatively low number of respondents. However, studies on residency application and PD’s surveys [25,31,32] with similar, or lower response rates have been previously published. With a response rate of 33.8%, this survey provides a limited glimpse into the PDs’ and APDs’ perspective on the value of anesthesiology LORs. The majority (80.65% [n = 50]) of our respondents have had 0–10 years of experience in their role. Despite these limitations, to the authors’ knowledge, this is the first study specifically examining the components of the LORs submitted for anesthesiology residency candidates.

Additionally, while the LOR is only one aspect of the application, and other information about the residency candidate may be obtained from other parts of the application such as the MSPE letter, the LOR may grow to play a bigger role into providing insight about right anesthesiology residency candidates.

Another limitation of our study is that letter-writers were not surveyed. It may be beneficial to survey the viewpoint of letter-writers on choice of words or adjectives and intended message. This may help improve the quality and formatting of LORs, thereby allowing for clear communication with residency programs regarding the residency candidates.

Furthermore, our survey only took into account words that either indicate a strong letter of recommendation or a weak letter of recommendation as binary descriptors. However, there may also be neutral words that neither indicate a strong or weak letter, which we did not investigate in our study.

Finally, survey data were collected anonymously, and consequently, responses from PDs could not be separated from those of APDs. Future studies may attempt to include a larger number of respondents, and to differentiate the perspective of PDs from that of APDs.

## Conclusion

LORs provide unique information about the anesthesiology residency candidates, not found elsewhere in the application. We found that LORs from anesthesiologists are most preferred and are most valuable to anesthesiology PDs and APDs. We have found that particular phrases and keywords are of much higher value to PDs and APDs when they include superlatives and ranking of the candidate. Narrative LORs are preferred by our respondents as compared to SLORs. Given the ‘coded’ language limitations to the current LOR recognized by our respondents, we propose several changes to the LOR format and process. The interpretation and objectivity of LORs may be improved upon with the addition of a Likert scale, addition of an anesthesiology-specific style guide and formalized training of the letter-evaluators.

At the core, the LOR is a form of communication between colleagues. Communication in the clinical setting is crucial and has been one of the single largest targets for improvement in healthcare delivery, efficacy, and efficiency[33]. The same concept may be applicable to LORs.

## Supplementary Material

Supplemental Material

## References

[1] Perkins JN, Liang C, McFann K, et al. Standardized letter of recommendation for otolaryngology residency selection. Laryngoscope. 2013;123(1):123–8. 23172646 10.1002/lary.23866 PMC3643334

[2] Fortune JB. The content and value of letters of recommendation in the resident candidate evaluative process. Current Surgery. 2002;59(1):79–83. 16093109 10.1016/s0149-7944(01)00538-4

[3] Oliveira GSD, Akikwaka T, Kendall MC, et al. Factors affecting admission to anesthesiology residency in the USA. Survey of Anesthesiology. 2012;56(6):280. 10.1097/ALN.0b013e31825fb04b 22739761

[4] Kaffenberger JA, Mosser J, Lee G, et al. A retrospective analysis comparing the new standardized letter of recommendation in dermatology with the classic narrative letter of recommendation. J Clin Aesthet Dermatol. 2016;9(9):36–42. PMC5110327 27878060

[5] Greenburg A, Doyle J, Mcclure D. Letters of recommendation for surgical residencies: what they say and what they mean. J Surg Res. 1994;56(2):192–198. 8121177 10.1006/jsre.1994.1031

[6] Kim S, George P. The relationship between preclinical grading and usmle scores in us allopathic medical schools. Fam Med. 2018;50(2):128–131. 29432628 10.22454/FamMed.2018.145163

[7] InCUS. (2019). Retrieved 2020 816, from https://www.usmle.org/incus/

[8] Chapman BV, Rooney MK, Ludmir EB, et al. Linguistic biases in letters of recommendation for radiation oncology residency applicants from 2015 to 2019. https://www.ncbi.nlm.nih.gov/pmc/articles/PMC7591242/. Published 10 27, 2020. Accessed 2021 24 10.1007/s13187-020-01907-x PMC7591242 33111188

[9] FREIDA residency program database: medical fellowship database. https://freida.ama-assn.org/Freida/. Accessed 7 2019.

[10] Accreditation Data System (ADS). https://apps.acgme.org/ads/Public/Reports/Report/1. Accessed 7 2019.

[11] Garmel GM, Grover CA, Quinn A, et al. Letters of Recommendation. J Emerg Med. 2019;57(3):405–410. 31375370 10.1016/j.jemermed.2019.04.020

[12] National resident matching program, data release and research committee: results of the 2018 nrmp program director survey. Washington, DC: National Resident Matching Program; 2018.

[13] Rajesh A, Rivera M, Asaad M, et al. What are we REALLY looking for in a letter of recommendation? J Surg Educ. 2019;76(6):008. 10.1016/j.jsurg.2019.06.008 31302033

[14] Naples R, French JC, Lipman JM. Best practices in letters of recommendation for general surgery residency: results of expert stakeholder focus groups. J Surg Educ. 2020;77(6):6. 10.1016/j.jsurg.2020.06.036 32651119

[15] Wright SM, Ziegelstein RC. Writing more informative letters of reference. J Gen Intern Med. 2004;19(5):588–593. 15109330 10.1111/j.1525-1497.2004.30142.x PMC1492321

[16] Friedman RB. Sounding board. Fantasy land. N Engl J Med.. 1983;308(11):651–653. 6828098 10.1056/NEJM198303173081110

[17] Reghunathan M, Mehta I, Gosman AA. Improving the standardized letter of recommendation in the plastic surgery resident selection process. J Surg Educ. 2020. DOI:10.1016/j.jsurg.2020.09.005 32994157

[18] Kominsky AH, Bryson PC, Benninger MS, et al. Variability of ratings in the otolaryngology standardized letter of recommendation. Otolaryngol Head Neck Surg. 2016;154(2):287–293. 26759426 10.1177/0194599815623525

[19] Bajwa NM, Yudkowsky R, Belli D, et al. Validity Evidence for a residency admissions standardized assessment letter for pediatrics. Teach Learn Med. 2018;30(2):173–183. 29190140 10.1080/10401334.2017.1367297

[20] Lee AG, Golnik KC, Oetting TA, et al. Re-engineering the resident applicant selection process in ophthalmology: a literature review and recommendations for improvement. Surv Ophthalmol. 2008;53(2):164–176. 18348881 10.1016/j.survophthal.2007.12.007

[21] Shultz K, Mahabir RC, Song J, et al. Evaluation of the current perspectives on letters of recommendation for residency applicants among plastic surgery program directors. Plast Surg Int. 2012;2012:728981. 22570782 10.1155/2012/728981 PMC3335712

[22] Messner AH, Shimahara E. Letters of recommendation to an otolaryngology/head and neck surgery residency program: their function and the role of gender. Laryngoscope. 2008;118(8):1335–1344. 18596564 10.1097/MLG.0b013e318175337e

[23] Morgenstern BZ, Zalneraitis E, Slavin S. Improving the letter of recommendation for pediatric residency applicants: an idea whose time has come? J Pediatr. 2003;143(2):143–144. 12970622 10.1067/S0022-3476(03)00255-5

[24] Attri J, Mohan B, Chatrath V. et al. Anesthesiologist: the silent force behind the scene. Anesthesia: Essays and Researches. 2015;9(3):293. 10.4103/0259-1162.159775 PMC4683484 26712962

[25] How to apply. https://anesthesia.ucsf.edu/how-apply. Published 2018. Accessed 9 26, 2020.

[26] Keim SM, Rein JA, Chisholm C, et al. A standardized letter of recommendation for residency application. Acad Emerg Med. 1999;6(11):1141–1146. 10569387 10.1111/j.1553-2712.1999.tb00117.x

[27] Saudek K, Saudek D, Treat R, et al. Dear program director: deciphering letters of recommendation. J Grad Med Educ. 2018;10(3):261–266. 29946380 10.4300/JGME-D-17-00712.1 PMC6008019

[28] Unthsc.edu. 2020. [online] Available at: https://www.unthsc.edu/career-center/wp-content/uploads/sites/71/ERAS-Letter-of-Recommendations_Guidelines_Updated_All-1.pdf

[29] Kong JH, Steele LJ, Botham CM. Ten simple rules for writing compelling recommendation letters. PLoS Comput Biol. 2021;17(2):2. 10.1371/journal.pcbi.1008656 PMC7906297 33630854

[30] Chopra D, Joneja M, Sandhu G, et al. Reference letters for subspecialty medicine residency positions: are they valuable for decision-making? results from a Canadian study.BMC Med Educ. 2020 [Published 2020 107];20(1):350. 33028313 10.1186/s12909-020-02270-7 PMC7540432

[31] Chung KC, Lau FH, Kotsis SV, et al. Factors influencing residents’ decisions to pursue a career in hand surgery: a national survey. J Hand Surg Am. 2004;29(4):738–747. 15249103 10.1016/j.jhsa.2004.04.009

[32] George LC, O’Neill R, Merchant AM. Residency training in robotic general surgery: a survey of program directors. Minim Invasive Surg. 2018;2018:1–7. 10.1155/2018/8464298 PMC5964613 29854454

[33] Vermeir P, Vandijck D, Degroote S, et al. Communication in healthcare: a narrative review of the literature and practical recommendations. Int J Clin Pract. 2015;69(11):1257–1267. 26147310 10.1111/ijcp.12686 PMC4758389

