# Supplementary material for: Attitudes towards and impact of letters of recommendation for anesthesiology residency applicants
Source: Med Educ Online. 2021 May 7;26(1):1924599. doi: 10.1080/10872981.2021.1924599 (PMC8118394; doi:10.1080/10872981.2021.1924599)
Supplement: Supplemental Material [file ZMEO_A_1924599_SM0435.docx]

|  | **Key Words indicating a strong LOR** | **Key Words indicating a weak LOR** |
| --- | --- | --- |
| **Personal characteristics** | accommodating | caring |
|  | adaptable | prompt |
|  | astute |  |
|  | attention to details |  |
|  | compassionate |  |
|  | dependable |  |
|  | focused |  |
|  | hardworking |  |
|  | passionate |  |
|  | persistent |  |
|  | professional |  |
| **Descriptors of clinical work attributes** | ability to handle critique | becomes rattled |
|  | acute processing of a rapidly changing medical condition | demonstrated improvement |
|  | chief resident material | needs reminding |
|  | excellent fund of knowledge | perform satisfactorily |
|  | good communication skills | tried hard |
|  | knows his patients | will help out if asked |
|  | performs at the level of a resident | was present |
|  | receptive to feedback |  |
|  | situational awareness |  |
|  | team player |  |
|  | technical skill progression |  |
| **Summative Superlatives** | best | adequate |
|  | enthusiastically | average |
|  | exceeds expectations | bottom _x% |
|  | excellent | eventually |
|  | exceptional | fine |
|  | exemplary | good |
|  | extraordinary | I recommend (without adverb) |
|  | highest | meets requirements |
|  | highest recommendation | non-traditional |
|  | highly | on par |
|  | refreshing to work with | please take a deeper look |
|  | remarkable | recommend to “your” program |
|  | stellar | solid |
|  | strong* | strong* |
|  | superb | superior |
|  | top _x% of student | very good |
|  | uplifting | we wish the applicant luck |
|  | valuable | will be a fine resident |
|  | valuable asset |  |
|  | we are recruiting |  |
|  | without reservation |  |
|  | would allow them to care for own family |  |

**Keywords suggested by respondents**

*strong may be considered good or bad according to respondents.
